# Uncovering the cathepsin system in heart failure patients submitted to Left Ventricular Assist Device (LVAD) implantation

**DOI:** 10.1186/s12967-014-0350-7

**Published:** 2014-12-12

**Authors:** Andrea D’Amico, Rosetta Ragusa, Raffaele Caruso, Tommaso Prescimone, Sandra Nonini, Manuela Cabiati, Silvia Del Ry, Maria Giovanna Trivella, Daniela Giannessi, Chiara Caselli

**Affiliations:** Scuola Superiore Sant’Anna, Institute of Life Sciences, 56100 Pisa, Italy; Laboratory of Cardiovascular Biochemistry, Institute of Clinical Physiology, Consiglio Nazionale delle Ricerche (CNR), Area della Ricerca – Via Moruzzi, 1, 56100 Pisa, Italy; Cardiovascular Department, Institute of Clinical Physiology, Consiglio Nazionale delle Ricerche (CNR), Niguarda Cà Granda Hospital, 20162 Milan, Italy; Cardiovascular Department, Niguarda Ca’ Granda Hospital, 20162 Milan, Italy

**Keywords:** Cathepsin system, Cardiac remodeling, Heart failure, LVAD therapy

## Abstract

**Background:**

In end-stage heart failure (HF), the implantation of a left ventricular assist device (LVAD) is able to induce reverse remodeling. Cellular proteases, such as cathepsins, are involved in the progression of HF. The aim of this study was to evaluate the role of cathepsin system in HF patients supported by LVAD, in order to determine their involvement in cardiac remodeling.

**Methods:**

The expression of cysteine (CatB, CatK, CatL, CatS) and serine cathepsin (CatG), and relative inhibitors (Cystatin B, C and SerpinA3, respectively) was determined in cardiac biopsies of 22 patients submitted to LVAD (pre-LVAD) and compared with: 1) control stable chronic HF patients on medical therapy at the moment of heart transplantation without prior LVAD (HT, n = 7); 2) patients supported by LVAD at the moment of transplantation (post-LVAD, n = 6).

**Results:**

The expression of cathepsins and their inhibitors was significantly higher in pre-LVAD compared to the HT group and LVAD induced a further increase in the cathepsin system. Significant positive correlations were observed between cardiac expression of cathepsins and their inhibitors as well as inflammatory cytokines. In the pre-LVAD group, a relationship of cathepsins with dilatative etiology and length of hospitalization was found.

**Conclusions:**

A parallel activation of cathepsins and their inhibitors was observed after LVAD support. The possible clinical importance of these modifications is confirmed by their relation with patients’ outcome. A better discovery of these pathways could add more insights into the cardiac remodeling during HF.

## Background

According to data from the American Heart Association (AHA) community surveillance component of the Atherosclerosis Risk in Communities (ARIC) study of the National Heart, Lung, and Blood Institute (NHLB1), the prevalence of HF will increase by 46% from 2012 to 2030, resulting in more than 8 million people ≥18 years of age with HF [1]. Implantation of left ventricular assist device (LVAD) has become a new gold standard to support end-stage HF (ESHF) patients as a bridge to heart transplantation [2]. LVAD support has been shown to affect myocardial remodeling, which is a complex pathologic process of ultrastructural rearrangement of the heart typically observed in chronic HF; it also sometimes promotes partial recovery of ventricular function, a process known as reverse remodeling [3]. In this process, alterations of the extracellular matrix (ECM) have a central role, and modulations of the activities of different proteases have become a topic of great interest [4]. Recent evidence supports the hypothesis that other proteases, such as calpains and cathepsins [5], may be involved in heart remodeling [4]. Among these, cathepsins have been the recent focus of several studies, owing to their emerging role in different diseases [6,7].

Since 1920, the term “cathepsin” stands for lysosomal-proteolytic enzyme regardless of the enzyme class [6]. Cathepsins were used as a measure of lysosomal activity and integrity but today they are believed to be implicated in the development and progression of cardiovascular disease [5]. This term includes serine proteases (cathepsins A and G), aspartic proteases (cathepsin D and E) as well as the better-known cysteine cathepsins (cathepsins B, C, F, H, K, L, O, S, V, X and W), as reported in Table 1. Cysteine cathepsins are typically known as housekeeping proteases essential for cardiac function; they are ubiquitous and contribute to distinct physiologic processes [5,7]. They are strictly regulated intracellularly by specific endogenous inhibitors belonging to the cystatin subgroup, cystatin A (CysA) and B (CysB) (also known as stefins A and B, respectively) and extracellularly by cystatin C (CysC) and kininogens [6,8,9]. Serine cathepsins are a key component of the inflammatory response as they are released from activated leukocytes and mast cells (MCs). The main representative cathepsin of this group is cathepsin G (CatG), which can modulate biological functions through the processing of chemokines, modulation of the cytokine network and the activation of specific cell surface receptors, especially in the heart [10]. CatG is regulated by the Serpin peptidase inhibitor A3 (SrpA3), a widely expressed member of the serpin superfamily, also released during the inflammatory response [11].

**Table 1:** **Cathepsin classification**

**Cathepsin**	**Cytosolic substrates**	**Function**	**Specific inhibitor**	**Ref**
*Serine protease*				
Cathepsin G	Protease activated receptors (PARs), elastin collagen, fibronectin,	Immune complex mediated inflammation, production of angiotensin II, degradation of ECM	SrpA3	[10-12]
Cathepsin A	Bioactive peptides (Endothelin 1, oxytocin, substance P, angiotensin 1)	Autophagy, elastic fiber formation, platelet activation		[13]
*Aspartic protease*				
Cathepsin D	Antiapoptotic molecules (Bid, Bax, caspase-8)	Protein degradation in an acidic milieu of lysosomes		[4,13,14]
Cathepsin E		Antigen presentation		[13]
*Cysteine proteases*				
Cathepsin B	Plasminogen, collagen, antiapoptotic molecules (Bcl-2, Bcl-xL, Mcl-1, and XIAP)	Protein catabolism, processing of antigens hormone activation and bone turnover	Cys C	[5,7,13-15]
Cathepsin C		Hydrolyze dipeptide esters, amides and beta-naphtylamides	Cys C	[13]
Cathepsin F	elastolytic activity	Antigen presenting	Cys C	[7,13]
Cathepsin H	elastolytic activity	Endopeptidase activity	Cys C	[5,13,16]
Cathepsin K	Elastin, collagen	Bone remodeling, elastolytic and collagenolytic activity	Cys C	[4,5,7,13,15-18]
Cathepsin L	Prohormones, MHC class II, trypsinogen, laminin fibronectin,, collagen	Keratinocyte differentiation, protein turnover, antigen presentation, elastolytic and collagenolytic activity	Cys C	[4,5,7,13,14,17]
Cathepsin S	Elastin, collagen, fibronectin, laminin, MHC class II	Protein degradation, elastolytic and collagenolytic activity, invariant chain II degradation	Cys C	[4,5,7,13,15,19]
Cathepsin V	elastolytic activity	Production of enkephalin and neuropeptide Y		[7,13,17]
Cathepsin W		Cell-mediated cytotoxicity		[13]
Cathepsin X		Phagocytosis, regulation of immune responses		[13]
Cathepsin Z		Protein degradation		[13]

Emerging evidence shows serine and cysteine cathepsin involvement in cardiac remodeling occurring in HF [5,17]. However, few data are available relative to cathepsin expression in cardiac tissue of ESHF patients and there are no reports on the effect of LVAD support in patients submitted to LVAD as a bridge to transplantation. The aim of this study was to evaluate the cathepsin system in cardiac tissue of ESHF patient candidates for LVAD implant compared to: 1. control stable chronic HF patients on medical therapy at the moment of heart transplantation without prior circulatory support; 2. patients supported by LVAD at the moment of transplantation. For this, expression of the main cysteine and serine cathepsins as well as their specific inhibitors was determined in cardiac tissue obtained from the three groups of ESHF patients.

## Materials and methods

### Patients and study design

Cardiac biopsies were obtained from ESHF patients supported by LVAD implant as a bridge to heart transplantation. Tissue samples were harvested from twenty-two patients at the moment of LVAD implantation from the apex of native heart (pre-LVAD group, n = 22). All these patients were supported by axial continuous-flow devices [16 were HeartMateII LVAD (Thoratec, Pleasanton, CA, USA), 4 were Incor LVAD (Berlin Heart AG),1 was De Bakey LVAD (MicroMed Technology Inc., Houston, TX, USA), and 1 was HeartWare LVAD (HeartWare International Inc., Framingham, MA, USA)].

In order to assess the effects of LVAD support on cathepsin systems the pre-LVAD group was compared with two other groups:

- A control group of seven ESHF patients supported by pharmacological therapy who directly went to heart transplantation without mechanical support. A total of thirty-five biopsies were collected at the moment of heart transplantation from pre-specified areas from the left ventricle (LV) (anterior basal, lateral basal and apex myocardial specimens) and from the right ventricle (RV) (anterior basal and the lateral basal myocardial specimens) as previously reported [20] (HT group, n = 35);
- A group of six patients supported by LVAD as bridge to transplantation. Cardiac biopsies (n = 30) were collected at the moment of the mechanical device explant from the same pre-specified areas of the control group [20]. Five patients were supported by axial continuous-flow devices [three were De Bakey LVADs (MicroMed Technology Inc.), one was a HeartMate II LVAF (Thoratec)] and one patient was supported by a pulsatile-flow device (NewCrTec, Rome, Italy). A total of thirty biopsies were collected at the moment of heart transplantation [20] (post-LVAD group, n = 30).

Immediately after collection, myocardial samples were frozen in liquid nitrogen and stored at −80°C until sample preparation.

Clinical parameters such as vital status and NYHA functional class were evaluated in all patients, both at admission and during LVAD support. In addition, the overall condition of multi-organ function was daily monitored according to the Sequential Organ Failure Assessment (SOFA). The SOFA system is a daily score from 0 to 4 assigned in proportion to the severity of functional deterioration for each of six individual organ systems (cardiovascular, respiratory, hepatic, renal, neurological, and hemocoagulative) [21]. The clinical course of these patients was assessed considering the following end-points: tSOFA score at 1 week, length of intensive care unit (ICU) stay, hospitalization, and 3-month survival. The combination of postoperative tSOFA score ≥11 and/or ICU death was taken into account as main composite adverse outcome during ICU stay.

#### Ethics statement

The study conformed the principles outlined in the Declaration of Helsinki and the study protocol was approved by Niguarda Cà Granda Hospital ethics committee (176/2005). All subjects gave written informed consent to participate to the study.

#### Inclusion and exclusion criteria for patient enrolment

Enrolment criteria for the LVAD implant were: idiopathic dilated/ischemic cardiomyopathy, not amenable to recovery by pharmacological or conventional surgical therapy; INTERMACS profile 1, 2 and 3 [22]; LVEF < 25%; peak oxygen consumption < 12 mL/Kg/min; body surface area > 1.5 m^2^; urgent heart transplantation not feasible; lack of contraindications for LVAD implantation; acceptable overall operative risk. Exclusion criteria were: irreversible renal/hepatic failure due to pre-existing chronic hepato-renal disease; severe diabetes mellitus with end-organ damage; severe peripheral vascular disease; coexisting active neoplasm; pregnancy; recurrent alcohol and drug abuse, and cognitive impairment severe enough to limit comprehension. As to the HT group, ESHF patients matched for age, sex, diagnosis and NYHA classes with pre-LVAD group were enrolled as control group.

### mRNA extraction and cDNA synthesis

Total RNA was extracted from heart samples with the use of the acid guanidiniumthiocyanate-phenol-chloroform method thanks to a Rneasy Midi kit (QiagenS.p.a, Milano, Italy) as described by the manufacturer. RNA concentration and purity were evaluated spectrophotometrically (BioPhotometer Eppendorf, Milan, Italy) and by electrophoresis of samples on Gel Star Stain (Lonza Rockland Inc., ME, USA) agarose gels. Only samples with spectrophotometric 260/280 nm ratios of 1.8–2.1 and clear 28S and 18S ribosomal RNA bands resulting from electrophoresis were used. A known amount of total RNA (Ambion, Inc., Austin, TX, USA) was used as marker. The RNA samples were stored at −80°C for use in gene expression studies.

Following DNAse treatment (RNase-Free DNase Set, QiagenS.p.A), first-stand cDNA was synthesized by IScript cDNA Synthesis Kit (Bio-Rad Laboratories, Hercules, CA, USA) starting from about 1 μg total RNA as template. Reverse transcriptase reaction sequence consisted of an incubation step at 25°C for 5 min, followed by three different cycles at 42°C for 30 min and 45–48°C for 10 min, in order to better separate the strands. The reverse transcriptase enzyme was inactivated by heating to 85°C for 5 min. The cDNA samples obtained were placed on ice and stored at 4°C for a maximum of 1 month.

### Real-time PCR

ProbeFinder 2.5 (Roche Applied Science) was used for designing primers. Real-time PCR reactions were performed in duplicate in the Bio-Rad C1000 thermal cycler (CFX-96 Real-Time PCR detection systems; Bio-Rad) using Eva-Green (SsoFASTEvaGreenSupermix; Biorad), a third-generation fluorophore, in order to monitoring cDNA amplification. PCR was performed in a volume of 20 μl/reaction; to minimize the influence of PCR inhibitors in real-time applications, all cDNA samples were diluted 1:10. Reaction mixture included 2 μl template cDNA (10 ng/ml), 0.2 mM of each primer (Sigma-Aldrich), 1X SsoFASTEvaGreenSuperMix (Bio-Rad), and sterile H_2_O. To assess product specificity, amplicons were systematically checked by melting curve analysis. Melting curves were generated from 65–95°C with increments of 0.5°C/cycle. Multiple inter-run calibrators were always used to allow comparison of Ct values obtained in different runs.

The reaction conditions of all primer pairs used were set out. In order to assess the optimal annealing temperature a gradient PCR was conducted while to verify efficiency a standard curve, obtained by scalar dilution of a cDNA pool (1:5, 1:25, 1:125, and 1:625), was always generated. We adhered to the Minimum Information for Publication of Quantitative real-time PCR Experiments (MIQEs) guidelines [23] to increase the reliability and integrity of study results and to promote efforts for experimental consistency and transparency between research laboratories.

The parameters derived from real-time PCR analysis required by MIQE guidelines are reported in Table 2.

**Table 2:** **Analytical details of gene primers for real-time PCR analysis**

		**Sequence**	**GenBank, accession #**	**Length (bp)**	**Temp (°C)**	**Efficiency (%)**	***R*** ^***2***^
**CAT-G**	Forward	TGACTGACTCTTCTTCTC	NM_001911.2	91	55	108.7	0.997
	Reverse	AGGAATTGGTTATTTATACTCT					
**CAT-B**	Forward	CTGTGGCAGCATGTGTGG	NM_001908.3	115	60	107.5	0.998
	Reverse	GCACCCTACATGGGATTCAT					
**CAT-L**	Forward	GGGAGGGCAGTTGAGGAC	NM_001912.4	111	64.5	98.5	0.995
	Reverse	GCAAGGATGAGTGTAGGATTCA					
**CAT-K**	Forward	GCCAGACAACAGATTTCCATC	NM_000396	75	60	108.1	0.999
	Reverse	CAGAGCAAAGCTCACCACAG					
**CAT-S**	Forward	GCTGAGGCACGAGATTCC	NM_004079	78	60	103.8	0.996
	Reverse	AGTCTCCACTCTGTCATCCA					
**CYS-B**	Forward	GAGTCCCCTCGCCAGATT	NM_000100.2	149	60	104	0.994
	Reverse	AACACAGGGAACTTCTTGTTTTCT					
**CYS-C**	Forward	AGGAGACAGACAGAGAAG	NM_000099.2	84	58	95.2	0.998
	Reverse	TATGAGAAGCAAGAAGGAA					
**SRPA3**	Forward	ACTCCAGACAGACGGCTTTG	NM_001085.4	73	60	100.5	0.995
	Reverse	ATTCTCTCCATTCTCAACTCTGC					
**YWHAZ**	Forward	ATGCAACCAACACATCCTATC	NM_00113572	178	60	95.3	0.997
	Reverse	GCATTATTAGCGTGCTGTCTT					
**PPIA**	Forward	CTTGGGCCGCGTCTCCTTCG	NM_021130	285	60	103.4	0.998
	Reverse	TTGGGAACCGTTTGTGTTTGGGGC					
**RPL13A**	Forward	CGCCCTACGACAAGAAAAAG	NM_012423	206	60	104.6	0.999
	Reverse	CCGTAGCCTCATGAGCTGTT					

### Data analysis

The geometric mean of the three most stably expressed genes (YWHAZ, RPL13A and PPIA) previous settled in our laboratory [20], was used for normalization of real-time PCR results.

The relative quantification was performed by ΔΔC_t_ method using Bio-Rad’s CFX96 manager software (CFX-96 Real-Time PCR detection systems, Bio-Rad Laboratories Inc.).

Data are expressed as mean and mean standard error (SEM). Variables were not normally distributed and were logarithmically transformed. Student’s t-tests (for comparisons between two groups) or ANOVA (for comparison of two or three groups) followed by Tukey post hoc tests were used to analyze the differences among groups. For correlation analysis, Spearman’s correlation was used to analyze the relationship between variables. A 2-tailed p-value <0.05 was considered statistically significant.

## Results

### Patient characteristics

Patient characteristics are reported in Table 3. Clinical features were compared according to the previously described experimental groups (pre-LVAD, HT and post-LVAD group).

**Table 3:** **Clinical features of ESHF patients according to sample groups**

	**Pre-LVAD (n = 22)**	**HT Patient (n = 7)**	P Value*	**Post-LVAD (n = 6)**	**P Value** ^**†**^
**Age, years**	58 (48–64)	55 (46–62)	0.459	44 (41–51)	0.031
**Male gender, n (%)**	19 (86)	5 (71)	0.569	6 (100)	1.000
**Etiology, n (%)**	-	-	0.202	-	0.673
**IDC**	12 (55)	6 (86)		45 (67)	
**IHD**	10 (46)	1 (14)		2 (33)	
**Treatments, n (%)**	-	-		-	
**ACE-I and/or ARB**	13 (59)	5 (71)	0.677	4 (67)	1.000
**Beta-blockers**	16 (80)	5 (71)	0.633	4 (67)	0.596
**Statins**	6 (27)	2 (29)	1.000	-	0.284
**Antiplatelet agents**	12 (54)	2 (29)	0.390	6 (100)^§^	0.062
**Inotropic support**	11 (50)	1 (14)	0.187	2 (33)	0.655
**Creatinine, mg/dL**	1.08 (0.9-1.53)	1.32 (1.00-1.78)	0.313	0.95 (0.83-1.48)	0.599
**t-Bil, mg/dL**	1.43 (0.55-1.90)	0.76 (0.48-1.14)	0.212	0.79 (0.62-1.35)	0.199
**NT-proBNP, ng/L**	2838 (1371–6042)	2389 (840–5762)	0.522	599 (158–1036)	0.007
**LVEF, %**	23 (19–25)	28 (20–29)	0.220	32 (20–33)	0.104
**LVEDV, mL**	202 (173–291)	228 (206–300)	0.185	237 (178–260)	0.820
**LVEDD, mm**	67 (57–71)	70 (68–79)	0.132	68 (60–75)	0.633
**RAP, mmHg**	5 (3–10)	3 (2–5)	0.074	3 (2–6)	0.969
**PCWP, mmHg**	25 (17–31)	11 (4–20)	0.019	10 (3–13)	0.023
**CI, L/min/m** ^**2**^	1.7 (1.4-2.2)	2.0 (1.5-2.7)	0.362	3.0 (2.2-3.3)	0.085
**PAPs, mmHg**	55 (42–63)	28 (19–42)	0.012	29 (21–33)	0.006

Data are expressed as median (25th-75th percentile) or frequency (percentage). ACE, angiotensin converting enzyme; ARB; angiotensin receptor blockers; CI, cardiac index; IDC, idiopathic dilated cardiomyopathy; IHD, ischemic heart disease; LVEDD, left ventricular end-diastolic diameter; LVEDV, left ventricular end-diastolic volume; LVEF, left ventricular ejection fraction; PAPs: pulmonary systolic arterial pressure; PCWP, pulmonary capillary wedge pressure; RAP, right atrial pressure; t-Bil, total bilirubin; P Value* pre-LVAD group vs HT group; P Value^†^ pre-LVAD group vs post-LVAD group; ^§^P <0.05 vs HT group.

#### Pre-LVAD and HT group

Median age of LVAD candidates (pre-LVAD group) was comparable to that of patients who underwent elective HT on medical therapy, without prior circulatory support (HT group). Idiopathic dilatative cardiomyopathy (IDC) was prevalent in both groups. Echocardiographic parameters as well as medical therapies did not differ between pre-LVAD and HT patients; anti-platelet and anti-coagulant agents, which were mandatory in pre-LVAD patients, were prevalent in pre-LVAD group. Total bilirubin and creatinine values did not show differences between the pre-LVAD group and HT group.

#### Pre- and post-LVAD patients prior to heart transplantation

Among the post-LVAD group, the median support time prior to heart transplantation was 367 (152–483) days. Median age of patients of post-LVAD group was lower than that of patient from pre-LVAD group. At heart transplantation, in patients of post-LVAD group, the levels of cardiac index, right atrial pressure, pulmonary capillary wedge pressure, as well as of NT-proBNP were lower than those of the pre-LVAD group, and comparable to those of HT group patients.

#### Postoperative LVAD outcome

After LVAD implantation, all pre-LVAD patients experienced postoperative hemodynamic improvement with respect to that at pre-implant (data not shown). At 3 postoperative months, 4 out of 22 (18%) pre-LVAD patients had died, in particular during ICU stay (second and third postoperative week), with multi-organ failure syndrome (MOFS) as main cause of death. Among survivors, the ICU length of stay was 14 [9-12,15-18,21-27] days, while hospitalization was of 45 (30–67) days. In all patients, the tSOFA score at 1 postoperative week was higher than that at pre-implant [9 (4–10) and 4 (2–5), respectively, p = 0.001]. However, eight patients experienced severe multi-organ failure evidenced by postoperative tSOFA score ≥11. Overall, nine out of 22 patients (41%) experienced postoperative tSOFA score ≥11 and/or ICU-death, together considered as composite critical outcome.

### Cathepsin expression in myocardial samples

**Figure 1:**
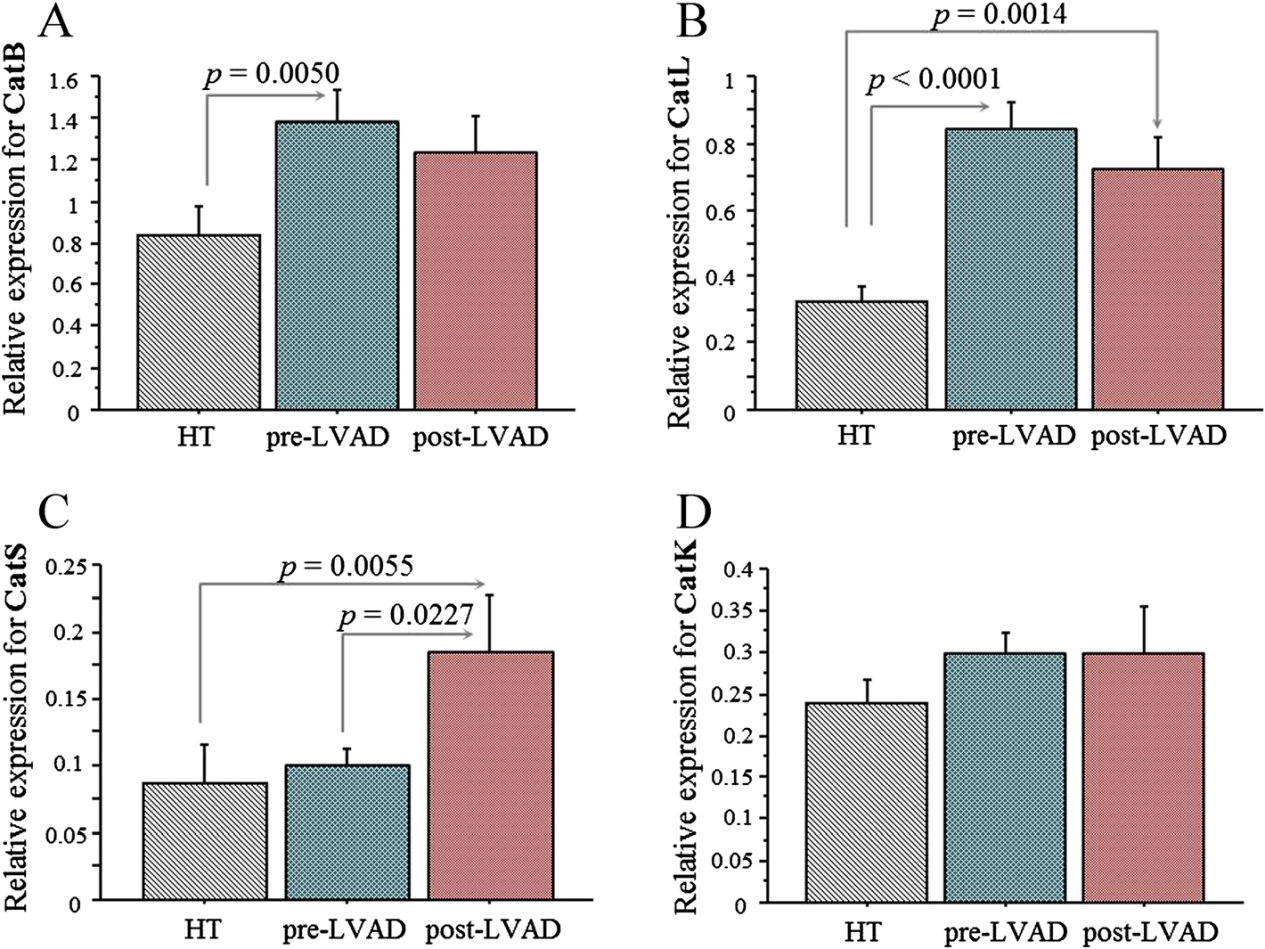
**Cysteine cathepsins.** mRNA expression of cysteine cathepsins in cardiac tissue from ESHF patient of pre-LVAD group (n=22), HT control group (n=35) and post-LVAD group (n=30), respectively. Relative expressions (mean value ± SEM) of CatB **(A)**, CatL **(B)**, CatS **(C)** CatK **(D)** are shown.

**Figure 2:**
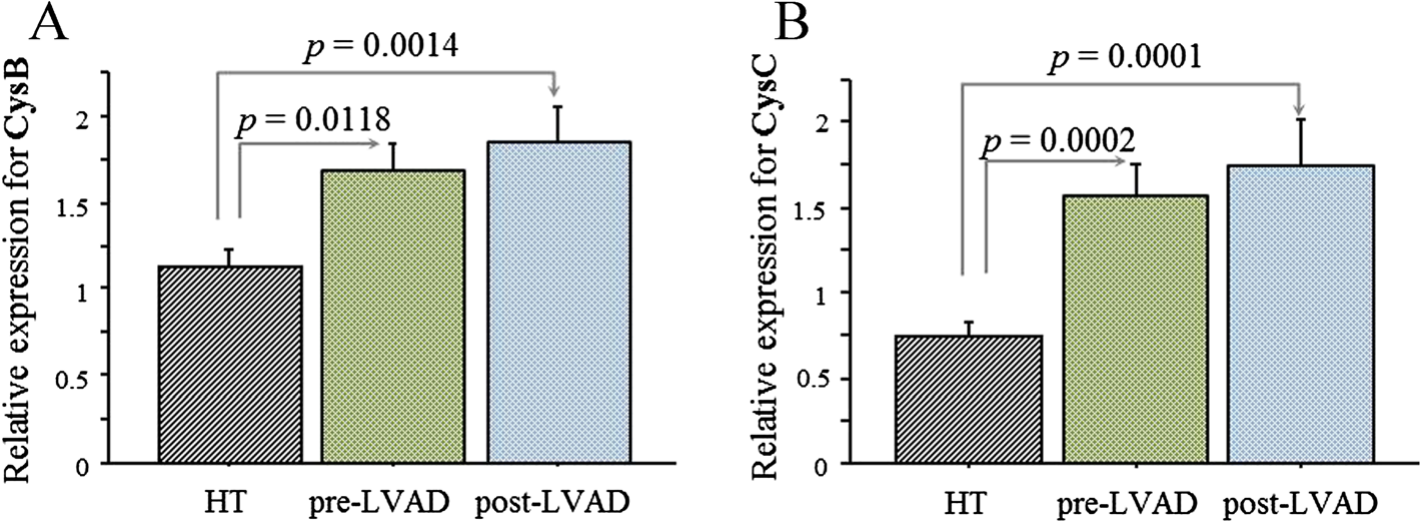
**Cysteine cathepsin inhibitors.** mRNA expression of cysteine cathepsin inhibitors in cardiac tissue from ESHF patient of pre-LVAD group (n = 22), HT control group (n = 35) and post-LVAD group (n = 30), respectively. Relative expression (mean value ± SEM) of CysB **(A)** and CysC **(B)** are shown.

**Figure 3:**
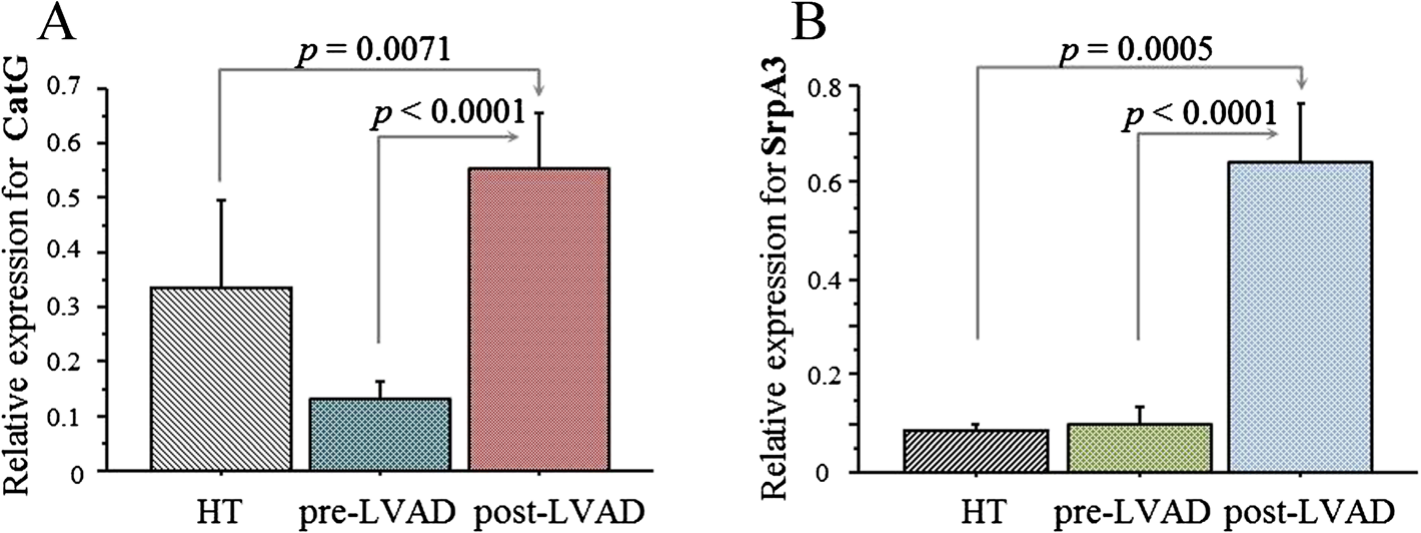
**Serine cathepsin system.** mRNA expression of serine cathepsin system in cardiac tissue from ESHF patients of pre-LVAD group (n = 22), HT control group (n = 35) and post-LVAD group (n = 30), respectively. Relative expressions (mean value ± SEM) of CatG **(A)** and SrpA3 **(B)** are shown.

The mRNA expression of cathepsins and inhibitors was compared according to the experimental groups, the pre-LVAD, HT and post-LVAD group and reported in Figures 1, 2, and 3.

#### At baseline

The cathepsin system was evaluated in myocardial specimens of ESHF patients at the moment of LVAD implantation (pre-LVAD group) and compared with a group of stable HF patients subjected to heart transplantation as control (HT group).

Cardiac mRNA expression of CatL resulted significantly higher in the pre-LVAD group than in the control group (HT group) (Figure 1). Similarly, the levels of the related cathepsin inhibitors CysB and CysC were significantly higher in the pre-LVAD group than in the HT group (Figure 2). Regarding serine cathepsins, both CatG and SrpA3 showed no significant variation in the pre-LVAD compared to HT group (Figure 3).

**Figure 4:**
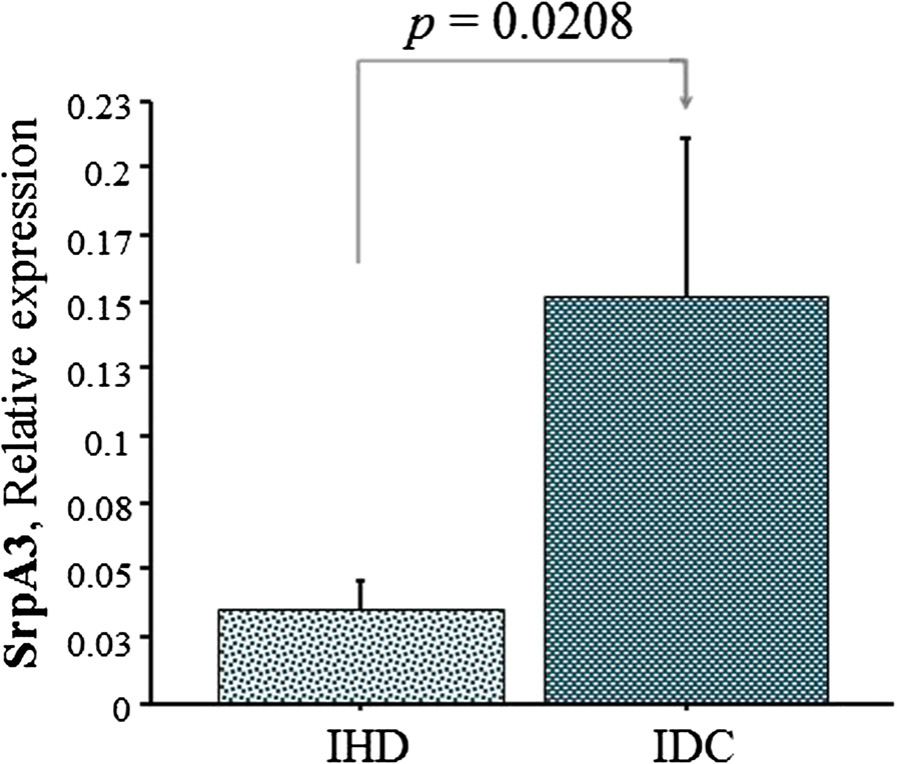
**SrpA3 and HF etiology in pre-LVAD patients.** IDC: Idiopathic dilatative cardiomyopathy; IHD: ischemic heart disease.

No correlation was observed between Cathepsin system and clinical characteristic of patients, including classical risk factors, echocardiographic parameters, and medications. As depicted in Figure 4, cardiac SrpA3 was able to identify the etiology of HF among clinical features of the pre-LVAD group, since they were significantly higher in IDC than IHD patients.

#### After LVAD implant

The effect of LVAD support on cathepsin systems was evaluated at tissue level by comparison of the pre-LVAD group with a group of patients at the time of heart transplantation (post-LVAD group). As for the cysteine cathepsin system, only CatS showed a significant increase in the post-LVAD compared with the pre-LVAD group (Figure 1) while in the serine cathepsin system both CatG and the inhibitor SrpA3 were significantly higher in post-LVAD (Figure 3). Cardiac mRNA transcripts of CatL, CatS, and CatG, and respective inhibitors resulted significantly higher in the post-LVAD group compared to their respective levels in the HT group (Figures 1, 2, and 3).

#### Relationship between cathepsins and inhibitors

Significant positive correlations were observed between the cardiac expression of cathepsins belonging to different classes and their specific inhibitors, as shown in Table 4. In particular, a strong correlation between CatS and CatG was observed; both of them showed again a strong relationship with the inhibitor SrpA3. A positive relation was observed between cysteine cathepsins, such as CatB, CatL and CatK, as well as their inhibitors (CysC and CysB).

**Table 4:** **Correlation among members of cathepsin system**

	**CatB**	**CatL**	**CatK**	**CatS**	**CysC**	**CysB**	**CatG**	**SrpA3**
**CatB**	---	rho = 0.51	rho = 0.50	rho = −0.14	rho = 0.42	rho = 0.32	rho = −0.17	rho = 0.26
		p = 0.0006	p = 0.0009	ns	p = 0.0032	p = 0.027	ns	ns
**CatL**		---	rho = 0.46	rho = 0.12	rho = 0.38	rho = 0.43	rho = −0.01	rho = 0.15
			p = 0.0011	ns	p = 0.0057	p = 0.0019	ns	ns
**CatK**			---	rho = 0.26	rho = 0.35	rho = 0.29	rho = 0.24	rho = 0.41
				ns	p = 0.0128	p = 0.037	ns	p = 0.0031
**CatS**				---	rho = 0.44	rho = 0.36	rho = 0.73	rho = 0.68
					p = 0.0023	p = 0.01	p <0.0001	p < 0.0001
**CysC**					---	rho = 0.70	rho = 0.40	rho = 0.36
						p < 0.0001	p = 0.0038	p = 0.007
**CysB**						---	rho = 0.24	rho = 0.16
							ns	ns
**CatG**							---	rho = 0.62
								p < 0.0001
**SrpA3**								---

### Cathepsins and inflammatory markers

Classic inflammatory markers IL-6, IL-8 and TNF-α were determined in cardiac samples from HT- pre-LVAD and post-LVAD groups (Table 5), as previously reported [20,24]. mRNA expression of IL-6 and IL-8 were higher in the post-LVAD group compared to LVAD candidate and HT groups. TNF-α mRNA expression was higher only in pre-LVAD group compared to post-LVAD group.

**Table 5:** **mRNA expression levels of IL-6, IL-8 and TNF-α according to patient groups**

	**Pre-LVAD (n = 22)**	**HT Patient (n = 7)**	P Value*	**Post-LVAD (n = 6)**	**P Value** ^**†**^
**Interleukin-6**	0.038 (0.021-0.089)	0.016 (0.006-0.034)	0.0027	0.595 (0.141-0.706)	<0.0001
**Interleukin-8**	0.010 (0.007-0.015)	0.041 (0.008-0.164)	0.0267	0.478 (0.237-0.901)	<0.0001
**TNF-α**	0.165 (0.101-0.393)	0.109 (0.064-0.646)	0.981	0.468 (0.175-0.828)	0.0166

Data are expressed as median (25th-75th percentile).

P Value* pre-LVAD group vs HT group; P Value^†^ pre-LVAD group vs post-LVAD group.

Relationships between cathepsin system and inflammatory markers were reported in Table 6. Cathepsins and their inhibitors positively correlate with IL-6. Only the serine cathepsin CatG and its specific inhibitor SrpA3 correlated with IL-8. Conversely, cysteine cathepsins CatL and CatB showed a positive correlation with TNF-α.

**Table 6:** **Correlation between cathepsins and inflammatory markers**

	**CatS**	**CatL**	**CatB**	**CatG**	**CysB**	**CysC**	**SrpA3**
**IL-6**	rho = 0.43	rho = 0.37	rho = 0.32	rho = 0.30	rho = 0.42	rho = 0.39	rho = 0.58
	p = 0.003	p = 0.007	p = 0.025	p = 0.032	p = 0.002	p = 0.003	p < 0.0001
**IL-8**	rho = 0.21	rho = −0.07	rho = 0.02	rho = 0.28	rho = 0.22	rho = 0.21	rho = 0.47
	ns	ns	ns	p = 0.051	ns	ns	p = 0.001
**TNF-α**	rho = −0.20	rho = 0.30	rho = 0.44	rho = −0.10	rho = 0.25	rho = 0.16	rho = 0.15
	ns	p = 0.049	p = 0.007	ns	ns	ns	ns

### Cathepsins and outcome indices

**Figure 5:**
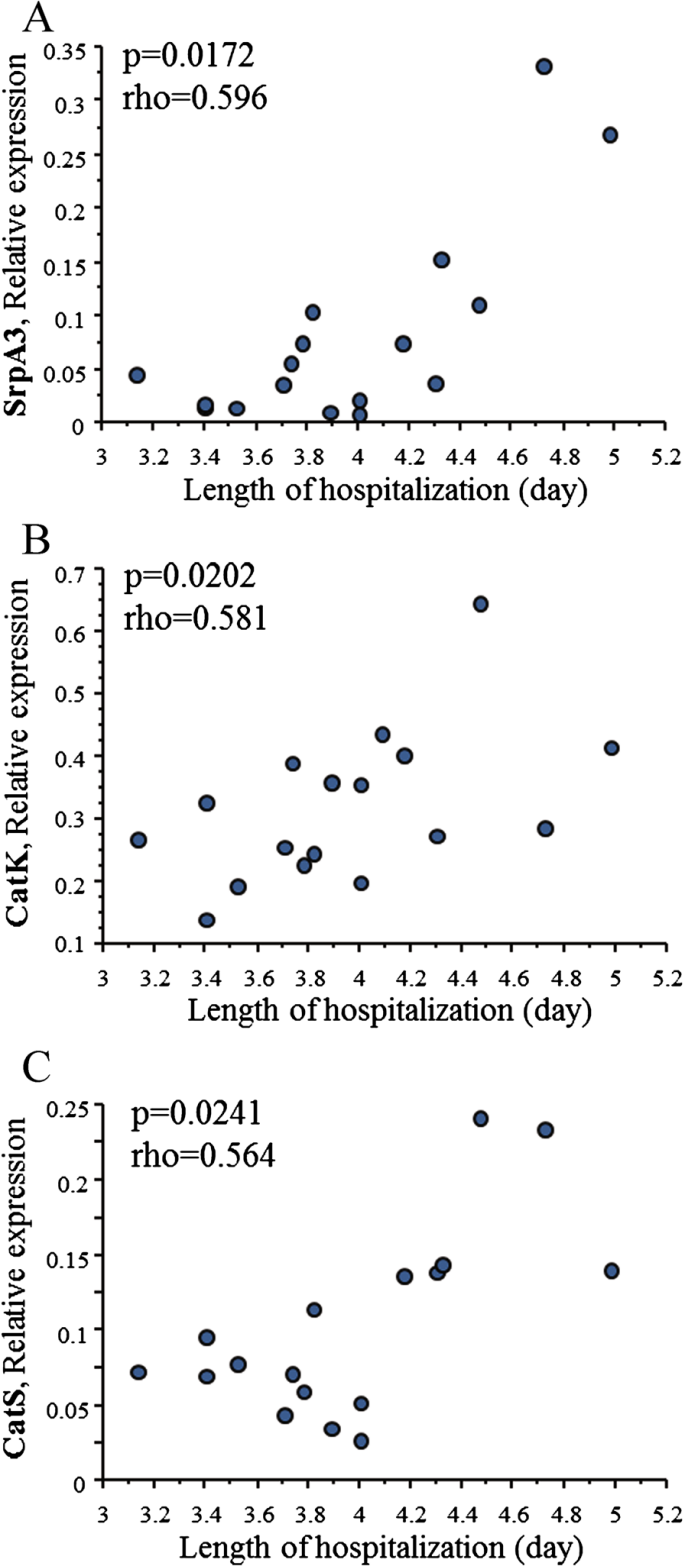
**Cathepsin system and clinical outcome.** Correlation between myocardial SrpA3 **(A)**, CatK **(B)** and CatS **(C)** and length of hospitalization in pre-LVAD patients.

The clinical course of ESHF (pre-LVAD group) was evaluated considering the outcome indices. CatS and CatK as well as SrpA3, assessed at pre-implant, showed a positive correlation with length of hospitalization (Figure 5). No significance was found with the other outcome indices.

## Discussion

This study shows for the first time that ESHF patient candidates for LVAD implant present higher expression levels of CatB and CatL as well as their specific inhibitors, CysB and CysC, compared to less severe HF patients undergoing medical therapy (HT group). In addition, modifications of cathepsin system, including CatS and CatG and its specific inhibitor, SrpA3, are influenced by mechanical unloading through LVAD support. A significant relationship with the length of hospitalization for CatS, CatK and SrpA3 at baseline was also observed, suggesting the relevance of this system regarding patient outcome.

In particular, before LVAD implantation CatL and CatB showed significantly higher mRNA levels compared to more stable patients who received transplants without LVAD support. These data are in tune with previous reports indicating that the cathepsin system is involved in cardiovascular function [5-7] and especially in HF progression [15,17] and hypertrophy [18]. Cheng XW et al. [15] shows that the expression of CatS and CatK was markedly increased throughout the myocardium of both rats and humans during HF, while only a low level of expression of these enzymes was observed in the myocardium of controls. Hua Y et al. [18], reported that in a knockout mice model the lack of CatK is associated to protective action inducing resistance to pressure overload–induced cardiac hypertrophy, fibrosis, and contractile anomalies. The mRNA expression levels of cysteine cathepsins, CatS, CatB, and CatK, increased in atherosclerotic plaque and in failing rat myocardium [16,25]. An important role in the regulation of apoptosis and immunoregulation was suggested for CatB [14]. Jiang H et al., report that CatK plays important roles in pathobiology of cardiovascular tissues in vivo and in vitro models [26]. Cathepsin inhibition also results in vascular cardioprotection via the reduction of inflammation and smooth muscle cell proliferation [14,27]. These evidences suggest an important involvement of cathepsin system in several molecular mechanisms underlying cardioprotective pathways.

At present, no data comparing cathepsin expression before and after LVAD support are available. Our results showed that the cathepsin cardiac system is activated after mechanical support. Different behaviour between both cysteine and serine cathepsins, and their specific inhibitors was observed after LVAD implant, pointing out a diverse pattern of expression of these classes of cathepsins. In particular, mRNA expression levels of cysteine cathepsins (CatB, CatL and CatK) and their specific inhibitors (CysB and CysC) were no modified after LVAD implant, while mRNA expression levels of serine cathepsin (CatG) and its specific inhibitor (SrpA3) showed a parallel significantly increase. Among cysteine catepsins, only CatS was modified by LVAD support. CatS appears to be involved in the development of various pathological conditions such as cardiovascular disease, obesity, and inflammatory diseases [19]. CatG is known to be a serine protease released from neutrophils [10] and MCs associated with inflammatory processes [28-31] and HF progression [31-33]. CatG concentration and activity were reported to decrease along with the reduction of inflammation and MC pro-inflammatory-type concentration [28]. After LVAD support, inflammatory cytokines remained significantly high [17,20,34] and the existence of a relationship between inflammatory cytokines and cathepsins has been shown in HF [5]. In vitro, mRNA expression of CatS, CatB, CatL, and CatK increase in neonatal cardiac myocytes in response to the inflammatory cytokines, identifying cardiac myocytes as a potential source of cathepsins [4]. TNF-α and IL-1β markedly cause the increase of cathepsin genes and protein expression in cardiomyocytes [5]. In agreement with these observations, in our study a positive correlation of cathepsins with inflammatory cytokines was observed, suggesting a possible influence of the inflammatory environment after mechanical support by LVAD on mRNA expression levels of cathepsins.

A recent hypothesis suggests that temporally regulated activation and suppression of inflammation may be critical for achieving effective cardiac repair and regeneration, indicating a paradoxical role for inflammation [34]. Similarly, some evidence points out the possible positive involvement of cathepsins in cardiac repair [17]. In our study mRNA expression profile of all cathepsins, except for CatK, was higher in patients supported by LVAD than HT group. In particular, CatL mRNA expression profile, which is known to be implicated in cardiac repair [5,35,36], was significantly higher in patients from pre- and post-LVAD groups than HT group, suggesting a possible involvement in cardiac remodeling.

In this study the cellular cathepsin specific inhibitors were also evaluated. The mRNA expression of CysC and CysB, the extracellular and intracellular cysteine cathepsin inhibitors respectively, resulted higher both before and after LVAD support compared to HT group in parallel with the increase of CatL. Decreased CysC expression is generally associated with an increased incidence of atherosclerosis and with severity of cardiovascular disease [37]. High plasma concentrations of CysC were independently associated with cardiovascular risk factors [8]. Moreover, in our study SrpA3, inhibitor of CatG, showed a significant increase in post-LVAD patients compared to pre-LVAD and HT patients and positively correlated with CatG and CatS. No data are available regarding SrpA3 involvement in HF especially in patients with mechanical support. SrpA3, mainly expressed in endothelial cells, is required for the regulation of several other proteases derived from MCs and neutrophils during the inflammatory response [11]. It is known that low SrpA3 expression levels were associated with an increased risk for atherosclerosis and aneurysm formation [12]. High mRNA expression of SrpA3 observed both after LVAD support and in IDC patients could suggest a possible involvement of this inhibitor in cardiac remodeling. As a matter of fact, the response of IDC to LVAD therapy is of particular interest because the myocardium is dysfunctional yet viable, unlike end-stage IHD [38]. The involvement of SrpA3 in cardiac remodeling is also supported by the positive correlations observed among cathepsin classes and their inhibitors. The strong correlation observed between cathepsins belonging to different classes, such as CatS and CatG, suggested the possible presence of synergic effects in cardiac remodeling. Cysteine and serine cathepsins are generally considered as two different systems: cysteine cathepsins are lysosome proteases having a role in cardiovascular remodeling, produced by cardiomyocytes, fibroblast and endothelial cells [5]; whereas serine proteases are typically known to be secreted by inflammatory cells such as neutrophils, macrophages and MCs [10].

Finally, in this study a significant positive correlation between CatS, CatK and SrpA3 with the length of hospitalization was found. These relationships with outcome could suggest a possible clinical relevance of the cathepsin system in HF. With regard to risk stratification in ESHF-patients, little is known about remodeling/inflammatory profiles and their impact on clinical outcome and prognosis, and it's reasonable to speculate a role of inflammatory system on the outcome of these fragile patients. The findings of this study underscore the importance to consider the remodeling/inflammatory parameters to deepen the knowledge of features of HF patients and better stratify the operative risk, and the risk of death after LVAD implantation [39].

### Study limitation

The main limitation of this study is the low number of patients. However, the internal control (HT group) and the post-LVAD group operated by collecting in the same patient myocardial tissue at HT time from both LV and RV allowed a better interpretation of the results in this limited sample size. Moreover, this low sample size made difficult to assess the impact of different clinical variables (i.e., therapies, risk factors, etc.) on the modulation of the cathepsin pathway. Due to the small size of myocardial biopsies, the activities of Cathepsin and their inhibitors could not be analyzed and, consequently, functional consequences of their parallel increase could not be deduced. Previous studies demonstrated that several cardiac drugs could have inhibitory effects on CatS and CatK expression in cardiovascular-renal tissues [40,41]. In this study, the lack of differences in pharmacological treatments among patient groups (Table 1) minimized possible effects of drugs on expression of cathepsins and inflammatory mediators.

## Conclusions

This study takes advantage of cathepsin system evaluation in an in vivo setting represented by a human model of HF. Our data suggest a parallel activation of molecules promoting the detrimental effect of ECM degradation such as CatS and CatG, and molecules promoting a positive regulation of cardiac remodeling, such as cystatins and serpins. Their modifications were associated with the inflammatory environment occurring after the device implantation. Determination of the specific pathways in HF may be essential in order to discover novel therapeutic strategies. In particular, novel treatment options may include the use of specific inhibitors for processes involved in HF progression such as proteolytic activities. These data are still of pivotal importance for understanding the process induced by mechanical heart unloading. More studies are necessary to better clarify the role of Cathepsin system in reverse remodeling.
